# Supplementary material for: Using the linear references from the pangenome to discover missing autism variants
Source: Nat Commun. 2026 Jan 23;17:1681. doi: 10.1038/s41467-026-68378-4 (PMC12909954; doi:10.1038/s41467-026-68378-4)
Supplement: Supplementary file 1 — Supplementary Information [file 41467_2026_68378_MOESM1_ESM.pdf]

Using the linear references from the pangenome to discover missing autism variants

Supplementary Notes

Software List

Software, database resources and custom algorithms used in this study are available as follows:

AnnotSV[1]: v3.4

asap[2]: v1.0.0, https://github.com/EichlerLab/asap

BCFtools[3]: v1.20

BoostSV: v1.0, https://github.com/jiadong324/BoostSV

CADD score (GRCh37)[4]: v1.3, https://cadd.gs.washington.edu

CADD-SV score[5]: v1.1.2

Canu[6]: v2.1.1

Canvas[7]: v1.40.0.1613+master

Clair3[8]: v1.0.2

CNVnator[9]: v0.3.3

continuous-methylation: https://github.com/projectoriented/continuous-methylation

contiguous-X: https://github.com/projectoriented/contiguous-X

cuteSV[10]: v2.1.0

dbNSFP[11]: v4.8a

deepTools: v3.5.5, https://github.com/deeptools/deepTools

DeepVariant[12]: v1.4.0

Delly[13]: v1.2.6

DESeq2[14]: v1.50.0

Ensembl Variant Effect Predictor (VEP)[15]: v110.1

GATK[12]: v4.3.0.0

gnomAD[16]: v4.1.0, https://gnomad.broadinstitute.org

hifiasm[17]: v0.16.1

Integrated Genomics Viewer (IGV)[18]: v2.16.0

Isoquant[19]: v3.10.0

Jalview[20]: v2.11.4.1

Kanpig[21]: v0.3.1

Kraken2[22]: v2.1.3

LongPhase[23]: v1.7.2

MAFFT[24]: v7.525

Manta[25]: v.1.5.0

Merqury[26]: v1.3

Meryl[26]: v1.4

Methylink: v0.6.0, https://github.com/projectoriented/methylink

minimap2[27]: v2.28.0

Modkit: v0.3.1, https://github.com/nanoporetech/modkit

ntsm[28]: v1.2.1

Paragragh[29]: v2.4

PAV[30]: v2.3.4

pbmm2: v1.13.1, https://github.com/PacificBiosciences/pbmm2

PBSV: v2.9.0, https://github.com/PacificBiosciences/pbsv

RagTag[31]: v2.1.0

rustybam: v0.1.33, https://github.com/mrvollger/rustybam

SAMtools[3]: v1.16.1

Sawfish[32]: v0.12.4

Smoove: v0.2.5, https://github.com/brentp/smoove

Sniffles[33]: v2.2

Somalier[34]: v0.2.19

subseq[35]: v1.0, https://github.com/EichlerLab/subseq-smk

SVbyEye R package[36]

TRGT[37]: v1.4.1

Truvari[38]: v4.3.1

UCSC Genome Browser tracks[39]: https://genome.ucsc.edu

UCSC LiftOver[40]: https://genome.ucsc.edu

VerifyBamID[41]: v2.0.1

yak: v1.0 or commit f389bad, https://github.com/lh3/yak.git

Supplementary Figures

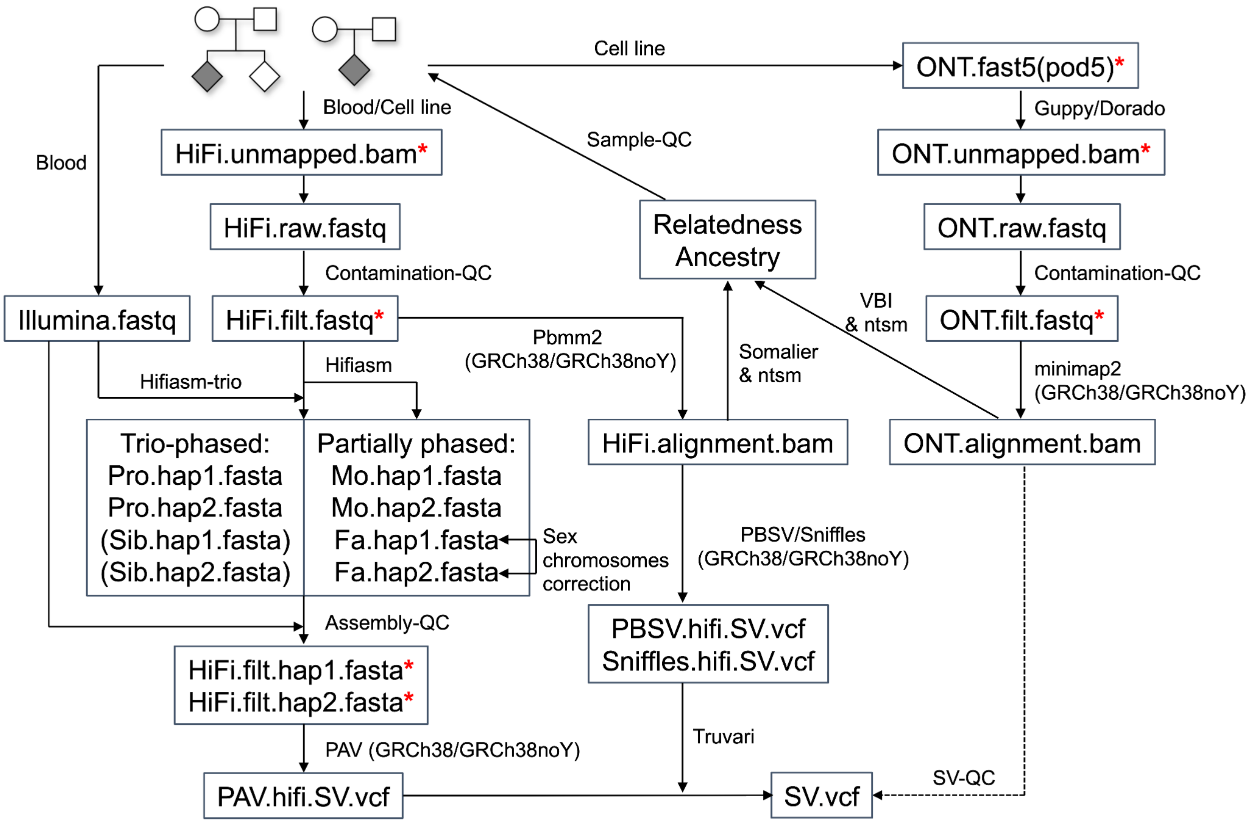

Supplementary Figure 1. Quality control and processing of long-read sequencing (LRS) data (Methods). Files marked with red asterisks are available in the SFARI Base under the Dataset ID DS0000104 and in the National Institute of Mental Health Data Archive (NDA) under the Collection ID 3780 (Data Availability).

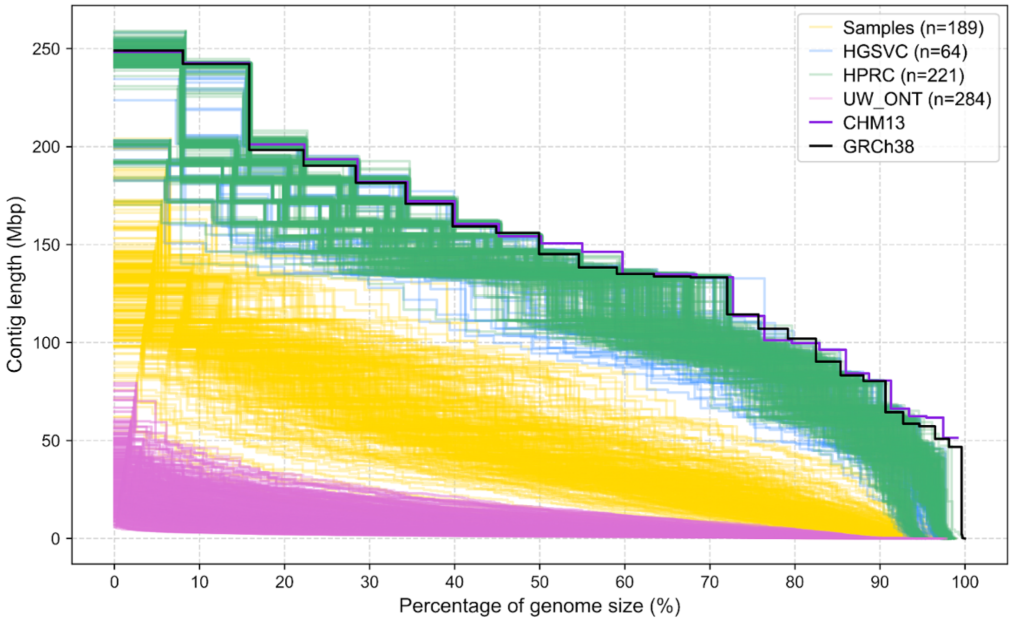

Supplementary Figure 2. NG(x) curves show cumulative assembly contiguity across sequencing batches. The NG(x) statistic represents the contig length at which x% of the estimated genome size is covered. Each color corresponds to a distinct sequencing batch: reference genome CHM13 (purple), GRCh38 (black), autism families (yellow), HGSVC controls (blue), HPRC controls (green), and 1KGP ONT controls (pink). Source data are provided in SFARI Base and the National Institute of Mental Health Data Archive (NDA) (Data Availability).

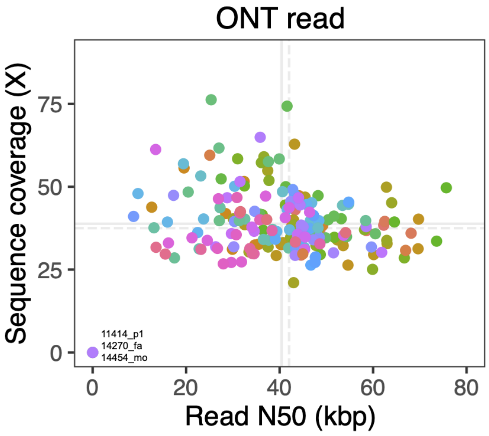

Supplementary Figure 3. ONT sequencing coverage and read N50 values in 51 unsolved families. Of the 189 individuals, 186 have ONT data available (members of the same family are color coded). Solid lines represent mean values, while dashed lines indicate median values. Source data are provided in Supplementary Data 1.

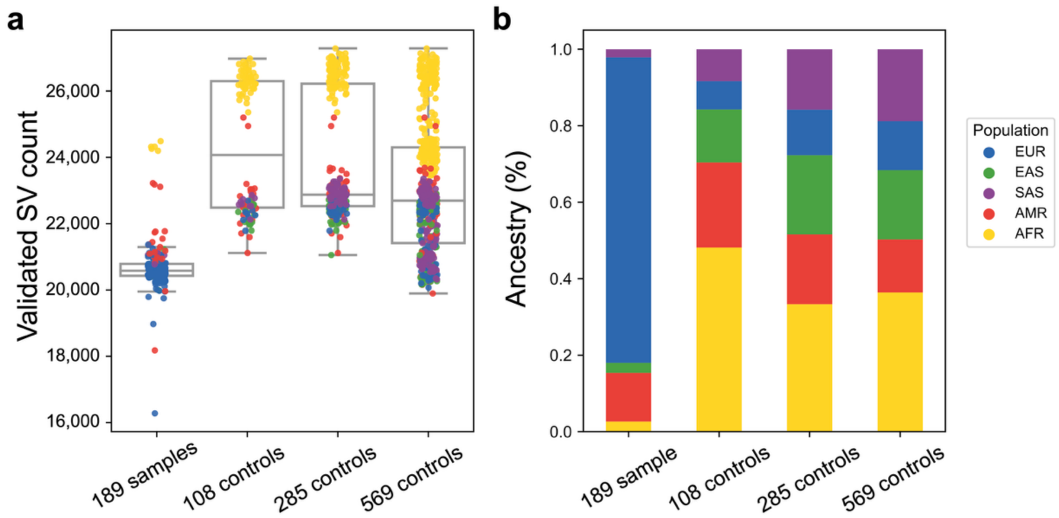

Supplementary Figure 4. Validated SV counts and ancestry composition in pangenome controls and 51 autism families. a SV counts in different cohorts. The 108 controls were from the HPRC and HGSVC. The 285 controls contain an additional 177 HPRC individuals. The 569 controls contain an additional 284 1KGP individuals. Box plots: center line represents the median; box limits indicate upper and lower quartiles; whiskers show 1.5× the interquartile range; individual points represent SV counts per sample. **b** Ancestry composition in different cohorts. The ancestry of 189 individuals was predicted by Somalier using HiFi alignments. Population groups were color-coded consistently across both figures. Source data are provided as a Source Data file.

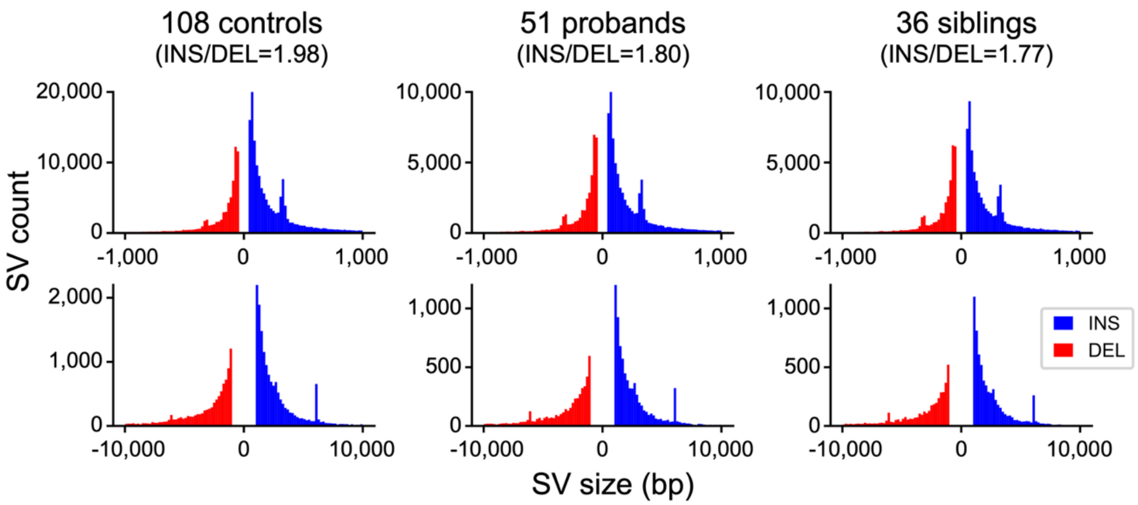

Supplementary Figure 5. Size distribution (bp) of nonredundant SVs in 108 controls, 51 probands and 36 siblings. The ratio of INS to DEL is marked in the parentheses. Source data are provided in Supplementary Data 2.

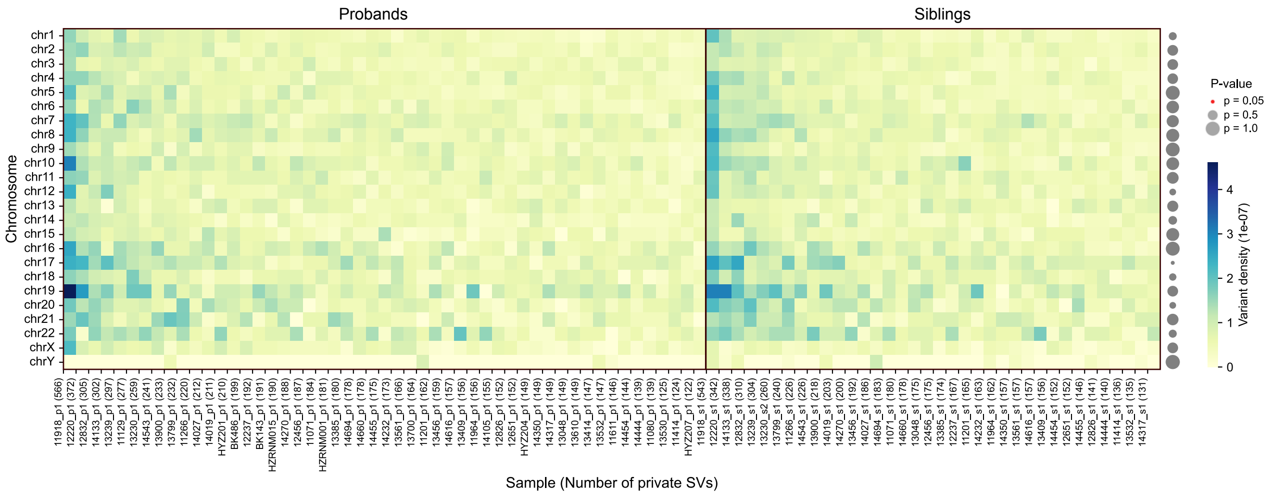

Supplementary Figure 6. Density heatmap (per chromosome) of private SVs from the 108-control set. P values on autosomes were calculated by χ^2 tests in 51 probands and 36 siblings, while sex chromosome analyses considered differences in copy number between sexes (92 and 51 X copies and 10 and 21 Y copies in probands and siblings respectively). Source data are provided in Supplementary Data 3 and Source Data.

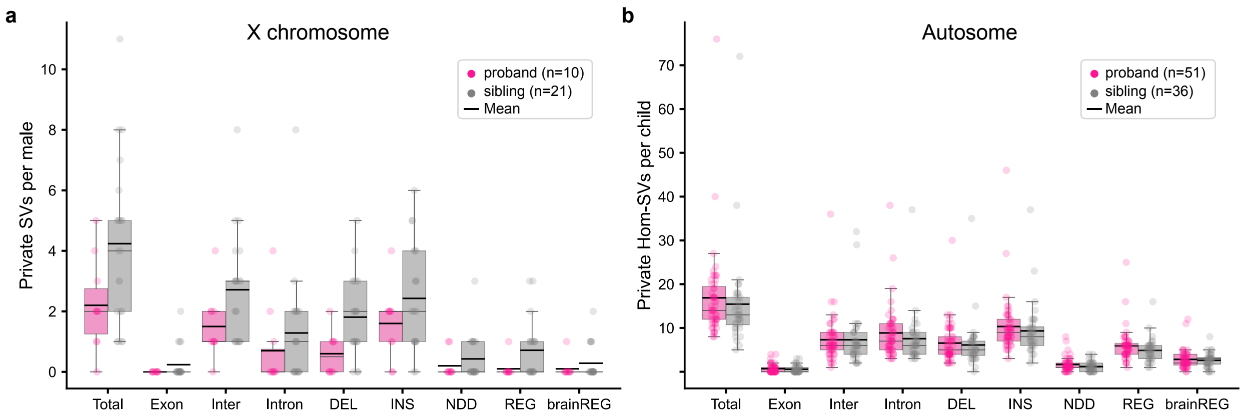

Supplementary Figure 7. Private SVs on X per male (a) and private homozygous SV (Hom-SV) on autosomes per child (b) from the 108-control set as a function of their categories, genomic location, SV types. The box plots compare private SV burden between probands (pink) and unaffected siblings (gray) for 51 probands (41 females and 10 males) and 36 unaffected siblings (15 females and 21 males). The center lines of the box plots represent the median; box limits indicate upper and lower quartiles; whiskers show 1.5× the interquartile range; individual points represent private SV counts per sample. The black line indicates the mean private count per sample. Source data are provided in Supplementary Data 3 and Source data.

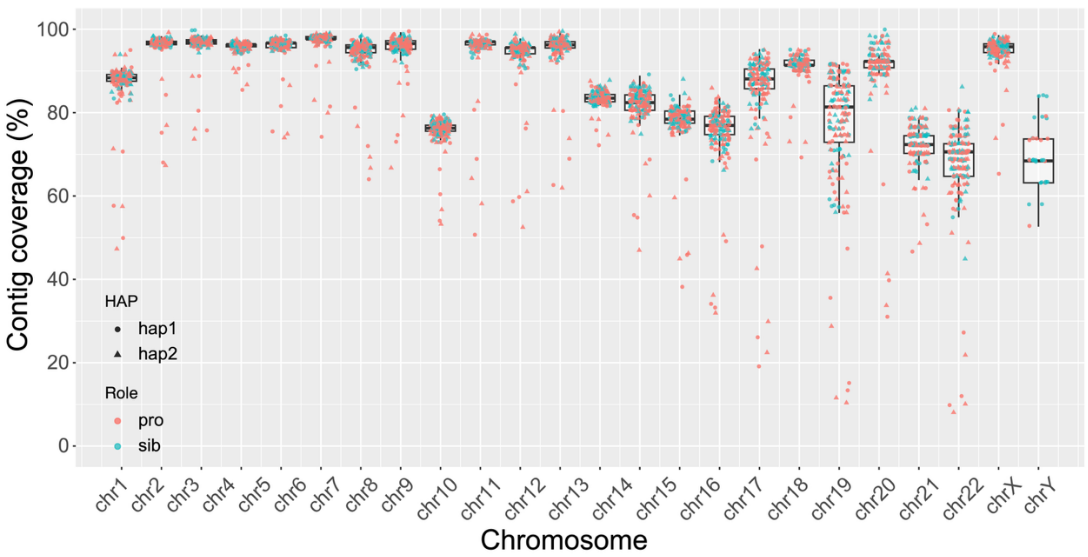

Supplementary Figure 8. Contig coverage relative to the T2T-CHM13v2.0 reference. Each reference chromosome was divided into 1 Mbp windows and we identified those covered by contigs that aligned to ≥95% of the window sequence with no more than three overlapping contigs. The coverage percentage was calculated as the number of qualified windows divided by the total number of windows, representing coverage relative to the reference chromosomes. Box plots: center line represents the median; box limits correspond to the first and third quartiles; whiskers show 1.5× the interquartile range. Each sample has an individual point per haplotype representing coverage. Source data are provided as a Source Data file.

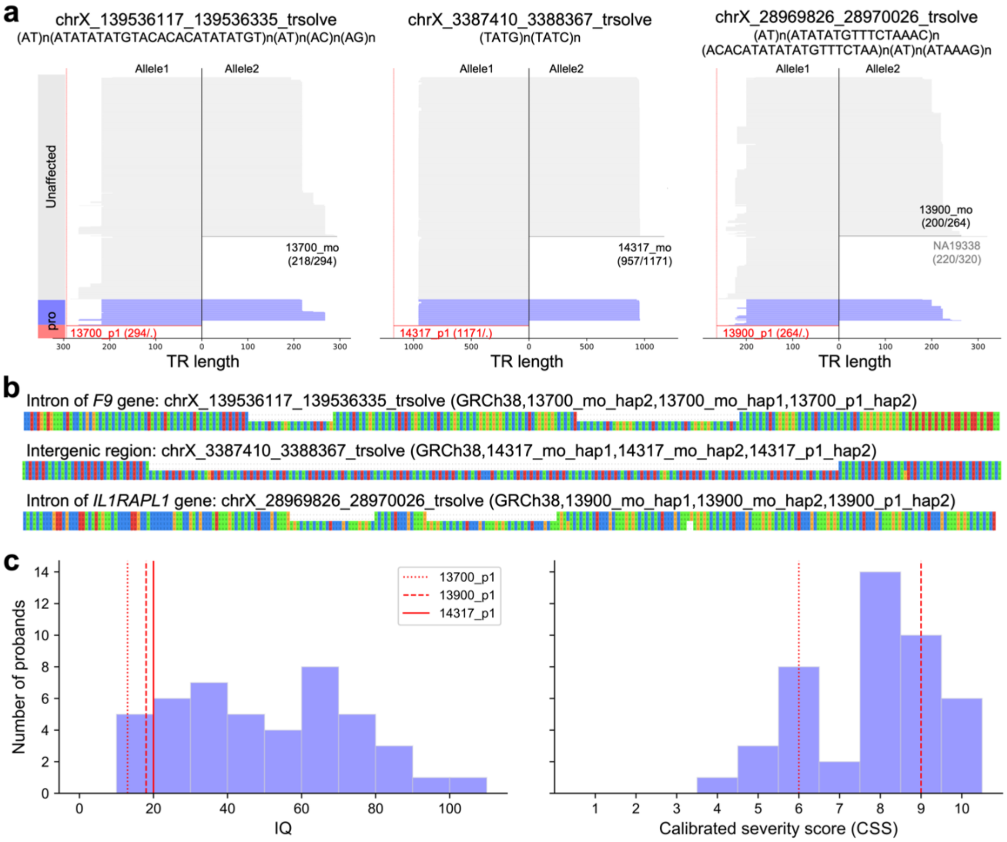

Supplementary Figure 9. Outlier TR expansions transmitted from mothers to male probands. a Allele lengths (generated by TRGT) of three TR catalogs[35] are shown in gray for unaffected individuals (285 controls and 138 unaffected samples) and in blue for affected probands. The three outlier expansions are highlighted in red and dark gray. **b** Transmission patterns from maternal haplotypes to male probands are illustrated using multiple sequence alignments (MSAs), with comparisons to the GRCh38 reference. Note that females, with two X chromosomes, display bidirectional TR lengths (both alleles), whereas males, with only one X chromosome, show unidirectional TR length from a single allele. **c** IQ and calibrated severity scores (CSS) of probands are shown (corresponding to Supplementary Data 1). Values for the three samples carrying outlier TR expansions on the X chromosome are indicated with red lines. Missing information denotes unavailable data. Source data are provided as a Source Data file.

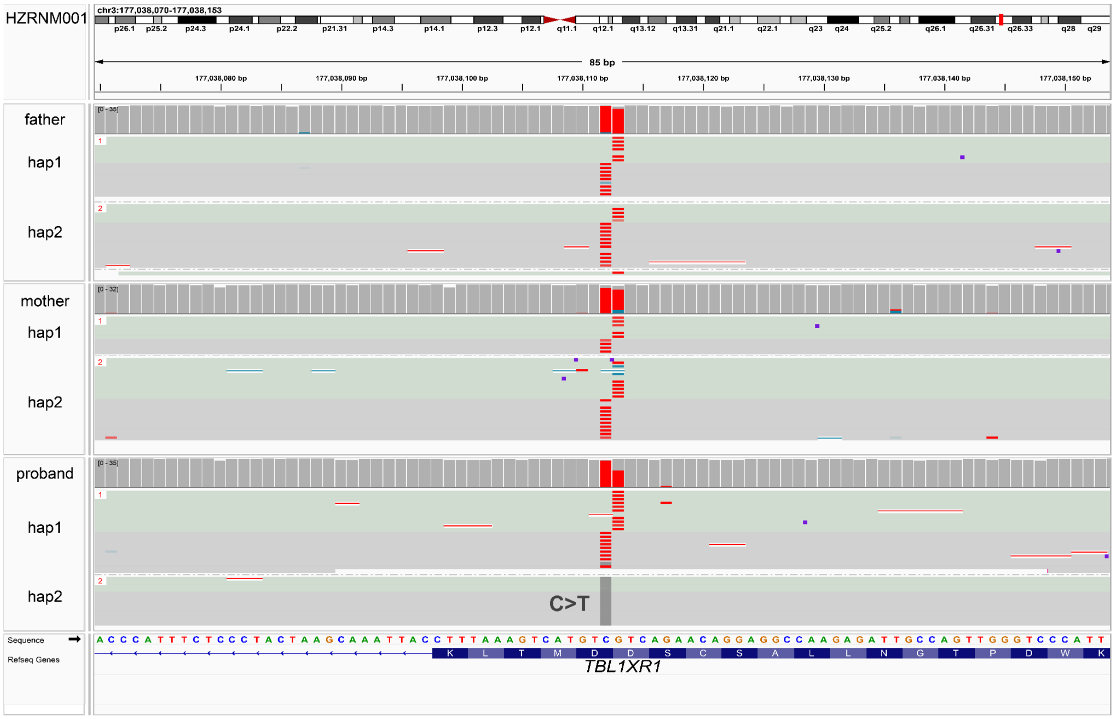

Supplementary Figure 10. Methylation changes at the CpG site induced by a DNM in *TBL1XR1*. Source data are provided in SFARI Base and the National Institute of Mental Health Data Archive (NDA) (Data Availability).

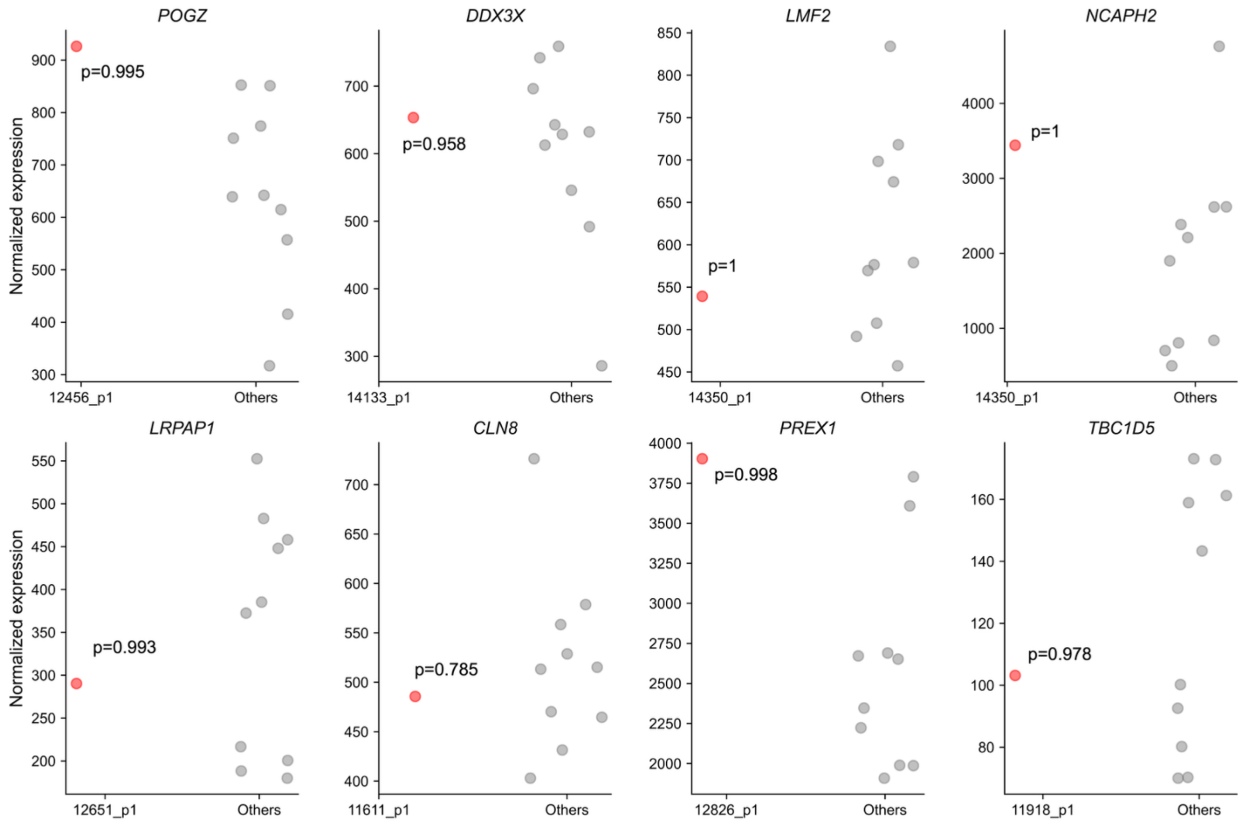

Supplementary Figure 11. Blood-derived long-read RNA sequencing (PacBio Kinnex) of seven candidate genes. Normalized read counts were generated using the counts() function from DESeq2 for 11 samples (including seven target probands and randomly selected probands and siblings from families 11201 and 14232). Adjusted p-values were calculated by comparing each target proband to the remaining samples using the Wald test in DESeq2. Source data are provided in SFARI Base, the National Institute of Mental Health Data Archive (NDA) (Data Availability), and the Source Data file.

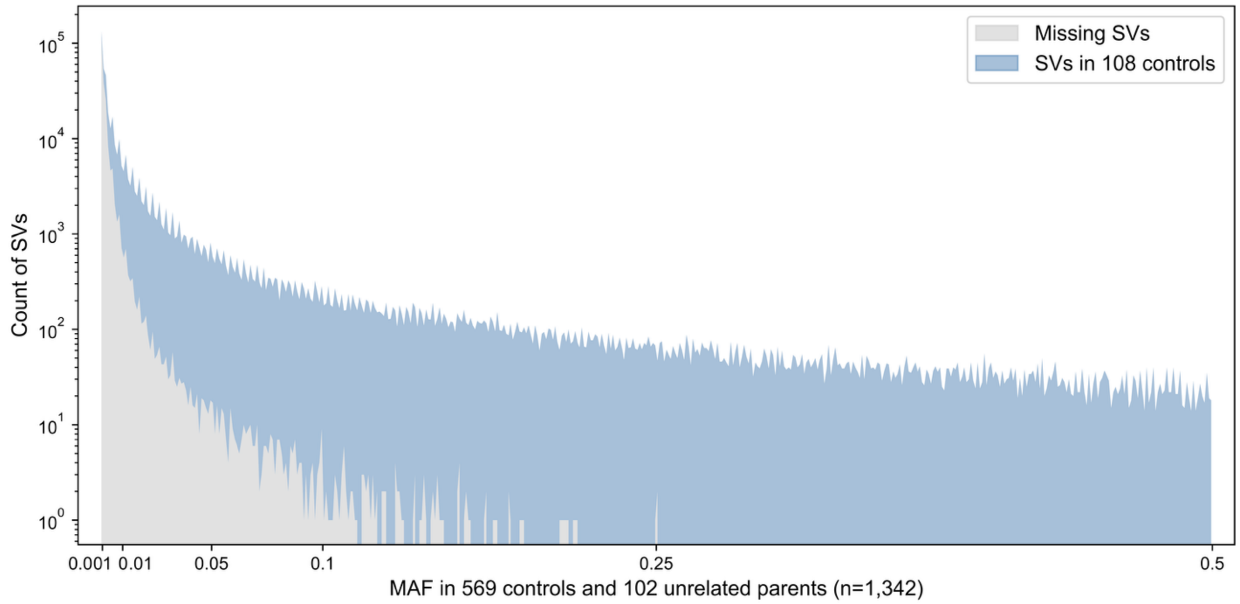

Supplementary Figure 12. Effect of control cohort size on SV discovery and minor allele frequency (MAF) spectrum. The plot shows the fraction of SVs captured (blue) in an initial control set of 108 genomes across different MAF thresholds derived from an expanded dataset comprising 569 controls and 102 unrelated parents (1,342 haplotypes in total). Only 16.2% of SVs with MAF < 0.1% in the larger dataset were detected in the smaller 108-control reference, underscoring the limited sensitivity of the smaller cohort for identifying ultra-rare variants. Source data are provided in Supplementary Data 7.

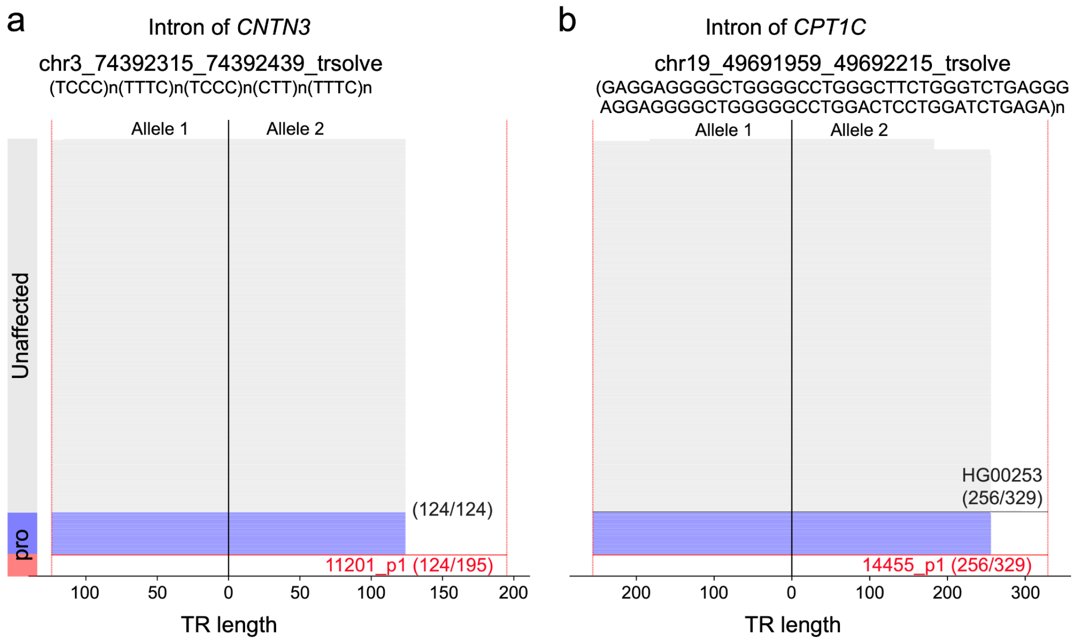

Supplementary Figure 13. TR expansions validated in larger control sets. The two *de novo* TR INS candidates were examined in the 285 controls using TRGT with HiFi alignments. The 71 bp INS (**a**, red) in the TR remains a distinct outlier even when compared to an expanded control set. However, the 73 bp INS (**b**, red) was seen in one control. TR length of unaffected individuals (285 controls and 138 unaffected samples) and probands were shown in gray and blue, respectively. Source data are provided as a Source Data file.

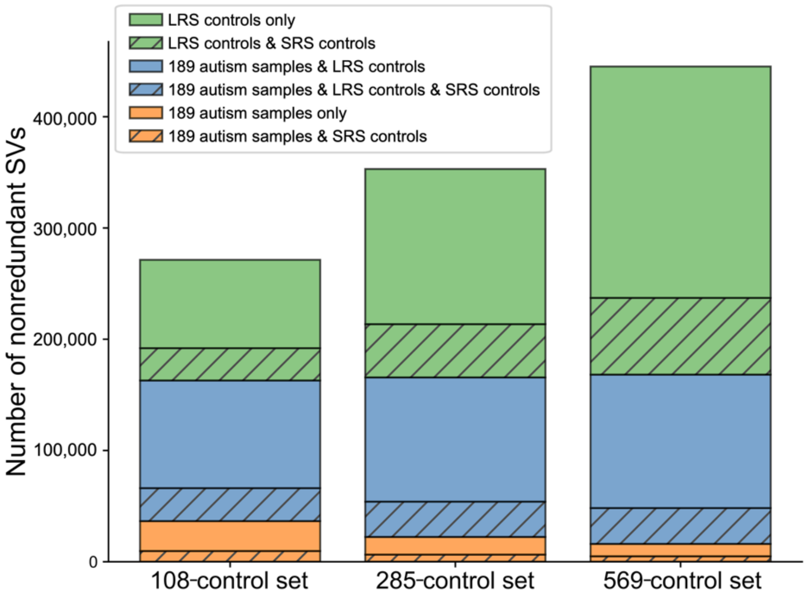

Supplementary Figure 14. Comparison of nonredundant SVs observed in LRS and SRS control sets. Collapsed nonredundant SVs from the three LRS control datasets were compared against SRS-based SVs (INS and DEL) generated by GATK-SV from 63,046 unrelated genomes in gnomAD v4.1 (corresponding to Supplementary Data 2, 5 and 7). Overlap between LRS and SRS SVs was defined using relaxed matching criteria in Truvari bench: a minimum of 50% reciprocal overlap, 50% size similarity, and maximum breakpoint distance of 500 bp, with no sequence similarity requirement. Cross-hatched bars indicate SVs that would have been detected by SRS. Source data are provided in Supplementary Data 2, 5, and 7.

Supplementary References

1. Geoffroy, V. *et al.* AnnotSV: an integrated tool for structural variations annotation. *Bioinformatics* **34**, 3572–3574 (2018).
2. Sui, Y. *et al.* Using the linear references from the pangenome to discover missing autism variants. *GitHub/Zenodo repository.* https://doi.org/10.5281/zenodo.18149644 (2026).
3. Danecek, P. *et al.* Twelve years of SAMtools and BCFtools. *GigaScience* **10**, giab008 (2021).
4. Kircher, M. *et al.* A general framework for estimating the relative pathogenicity of human genetic variants. *Nat Genet* **46**, 310–315 (2014).
5. Kleinert, P. & Kircher, M. A framework to score the effects of structural variants in health and disease. *Genome Res.* **32**, 766–777 (2022).
6. Koren, S. *et al.* Canu: scalable and accurate long-read assembly via adaptive k-mer weighting and repeat separation. *Genome Res.* **27**, 722–736 (2017).
7. Roller, E., Ivakhno, S., Lee, S., Royce, T. & Tanner, S. Canvas: versatile and scalable detection of copy number variants. *Bioinformatics* **32**, 2375–2377 (2016).
8. Zheng, Z. *et al.* Symphonizing pileup and full-alignment for deep learning-based long-read variant calling. *Nat Comput Sci* **2**, 797–803 (2022).
9. Abyzov, A., Urban, A. E., Snyder, M. & Gerstein, M. CNVnator: An approach to discover, genotype, and characterize typical and atypical CNVs from family and population genome sequencing. *Genome Res.* **21**, 974–984 (2011).
10. Jiang, T. *et al.* Long-read-based human genomic structural variation detection with cuteSV. *Genome Biology* **21**, 189 (2020).

11. Liu, X., Li, C., Mou, C., Dong, Y. & Tu, Y. dbNSFP v4: a comprehensive database of transcript-specific functional predictions and annotations for human nonsynonymous and splice-site SNVs. *Genome Medicine* **12**, 103 (2020).
12. Poplin, R. *et al.* A universal SNP and small-indel variant caller using deep neural networks. *Nat Biotechnol* **36**, 983–987 (2018).
13. Rausch, T. *et al.* DELLY: structural variant discovery by integrated paired-end and split-read analysis. *Bioinformatics* **28**, i333–i339 (2012).
14. Love, M. I., Huber, W. & Anders, S. Moderated estimation of fold change and dispersion for RNA-seq data with DESeq2. *Genome Biology* **15**, 550 (2014).
15. McLaren, W. *et al.* The Ensembl Variant Effect Predictor. *Genome Biology* **17**, 122 (2016).
16. Collins, R. L. *et al.* A structural variation reference for medical and population genetics. *Nature* **581**, 444–451 (2020).
17. Cheng, H., Concepcion, G. T., Feng, X., Zhang, H. & Li, H. Haplotype-resolved de novo assembly using phased assembly graphs with hifiasm. *Nat Methods* **18**, 170–175 (2021).
18. Robinson, J. T. *et al.* Integrative genomics viewer. *Nat Biotechnol* **29**, 24–26 (2011).
19. Prjibelski, A. D. *et al.* Accurate isoform discovery with IsoQuant using long reads. *Nat Biotechnol* **41**, 915–918 (2023).
20. Waterhouse, A. M., Procter, J. B., Martin, D. M. A., Clamp, M. & Barton, G. J. Jalview Version 2—a multiple sequence alignment editor and analysis workbench. *Bioinformatics* **25**, 1189–1191 (2009).

21. English, A. C., Cunial, F., Metcalf, G. A., Gibbs, R. A. & Sedlazeck, F. J. K-mer analysis of long-read alignment pileups for structural variant genotyping. *Nat Commun* **16**, 3218 (2025).

22. Wood, D. E., Lu, J. & Langmead, B. Improved metagenomic analysis with Kraken 2. *Genome Biology* **20**, 257 (2019).

23. Lin, J.-H., Chen, L.-C., Yu, S.-C. & Huang, Y.-T. LongPhase: an ultra-fast chromosome-scale phasing algorithm for small and large variants. *Bioinformatics* **38**, 1816–1822 (2022).

24. Katoh, K., Misawa, K., Kuma, K. & Miyata, T. MAFFT: a novel method for rapid multiple sequence alignment based on fast Fourier transform. *Nucleic Acids Research* **30**, 3059–3066 (2002).

25. Chen, X. *et al.* Manta: rapid detection of structural variants and indels for germline and cancer sequencing applications. *Bioinformatics* **32**, 1220–1222 (2016).

26. Rhie, A., Walenz, B. P., Koren, S. & Phillippy, A. M. Merqury: reference-free quality, completeness, and phasing assessment for genome assemblies. *Genome Biology* **21**, 245 (2020).

27. Li, H. New strategies to improve minimap2 alignment accuracy. *Bioinformatics* **37**, 4572–4574 (2021).

28. Chu, J., Rong, J., Feng, X. & Li, H. ntsm: an alignment-free, ultra-low-coverage, sequencing technology agnostic, intraspecies sample comparison tool for sample swap detection. *GigaScience* **13**, giae024 (2024).

29. Chen, S. *et al.* Paragraph: a graph-based structural variant genotyper for short-read sequence data. *Genome Biology* **20**, 291 (2019).

30. Ebert, P. *et al.* Haplotype-resolved diverse human genomes and integrated analysis of structural variation. *Science* **372**, eabf7117 (2021).
31. Alonge, M. *et al.* Automated assembly scaffolding using RagTag elevates a new tomato system for high-throughput genome editing. *Genome Biology* **23**, 258 (2022).
32. Saunders, C. T. *et al.* Sawfish: improving long-read structural variant discovery and genotyping with local haplotype modeling. *Bioinformatics* **41**, btaf136 (2025).
33. Smolka, M. *et al.* Detection of mosaic and population-level structural variants with Sniffles2. *Nat Biotechnol* **42**, 1571–1580 (2024).
34. Pedersen, B. S. *et al.* Somalier: rapid relatedness estimation for cancer and germline studies using efficient genome sketches. *Genome Medicine* **12**, 62 (2020).
35. Porubsky, D. *et al.* Human de novo mutation rates from a four-generation pedigree reference. *Nature* 1–10 (2025) doi:10.1038/s41586-025-08922-2.
36. Porubsky, D. *et al.* SVbyEye: a visual tool to characterize structural variation among whole-genome assemblies. *Bioinformatics* **41**, btaf332 (2025).
37. Dolzhenko, E. *et al.* Characterization and visualization of tandem repeats at genome scale. *Nat Biotechnol* **42**, 1606–1614 (2024).
38. English, A. C., Menon, V. K., Gibbs, R. A., Metcalf, G. A. & Sedlazeck, F. J. Truvari: refined structural variant comparison preserves allelic diversity. *Genome Biology* **23**, 271 (2022).
39. Perez, G. *et al.* The UCSC Genome Browser database: 2025 update. *Nucleic Acids Research* **53**, D1243–D1249 (2025).
40. Hinrichs, A. S. *et al.* The UCSC Genome Browser Database: update 2006. *Nucleic Acids Research* **34**, D590–D598 (2006).

41. Zhang, F. *et al.* Ancestry-agnostic estimation of DNA sample contamination from sequence reads. *Genome Res.* **30**, 185–194 (2020).
