# Supplementary material for: Using the linear references from the pangenome to discover missing autism variants
Source: Nat Commun. 2026 Jan 23;17:1681. doi: 10.1038/s41467-026-68378-4 (PMC12909954; doi:10.1038/s41467-026-68378-4)
Supplement: Supplementary file 2 — Description of Additional Supplementary Files [file 41467_2026_68378_MOESM2_ESM.pdf]

Description of Additional Supplementary Files

Supplementary Data 1-8:

Supplementary Data 1.1 - Sample list and sequencing metrics for 51 unsolved autism families (189 samples).
Supplementary Data 1.2 - Sample list of long-read controls.
Supplementary Data 1.3 - Summary of ancestry for controls and 189 autism samples.

Supplementary Data 2 - Summary of collapsed SVs from 108 long-read controls and 189 samples.

Supplementary Data 3 - Summary of rare SVs in 87 children filtered by 108 long-read controls.

Supplementary Data 4.1 - Summary of *de novo* mutations identified by long-read sequencing.
Supplementary Data 4.2 - Summary of pathogenic and candidate variants identified in this study.

Supplementary Data 5 - Summary of collapsed SVs from 285 long-read controls and 189 samples.

Supplementary Data 6 - Summary of rare SVs in 87 children filtered by 285 long-read controls.

Supplementary Data 7 - Summary of collapsed SVs from 569 long-read controls and 189 samples.

Supplementary Data 8 - Summary of rare SVs in 87 children filtered by 569 long-read controls.
