# Supplementary material for: Using the linear references from the pangenome to discover missing autism variants
Source: Nat Commun. 2026 Jan 23;17:1681. doi: 10.1038/s41467-026-68378-4 (PMC12909954; doi:10.1038/s41467-026-68378-4)
Supplement: Supplementary file 11 — Reporting Summary [file 41467_2026_68378_MOESM11_ESM.pdf]

nature portfolio

Corresponding author(s): Evan E. Eichler

Last updated by author(s): Dec 19, 2025

Reporting Summary

Nature Portfolio wishes to improve the reproducibility of the work that we publish. This form provides structure for consistency and transparency in reporting. For further information on Nature Portfolio policies, see our Editorial Policies and the Editorial Policy Checklist.

Statistics

For all statistical analyses, confirm that the following items are present in the figure legend, table legend, main text, or Methods section.

n/a	Confirmed	
☐	☒	The exact sample size (*n*) for each experimental group/condition, given as a discrete number and unit of measurement
☐	☒	A statement on whether measurements were taken from distinct samples or whether the same sample was measured repeatedly
☐	☒	The statistical test(s) used AND whether they are one- or two-sided<br>*Only common tests should be described solely by name; describe more complex techniques in the Methods section.*
☐	☒	A description of all covariates tested
☐	☒	A description of any assumptions or corrections, such as tests of normality and adjustment for multiple comparisons
☐	☒	A full description of the statistical parameters including central tendency (e.g. means) or other basic estimates (e.g. regression coefficient) AND variation (e.g. standard deviation) or associated estimates of uncertainty (e.g. confidence intervals)
☐	☒	For null hypothesis testing, the test statistic (e.g. *F*, *t*, *r*) with confidence intervals, effect sizes, degrees of freedom and *P* value noted<br>*Give P values as exact values whenever suitable.*
☒	☐	For Bayesian analysis, information on the choice of priors and Markov chain Monte Carlo settings
☒	☐	For hierarchical and complex designs, identification of the appropriate level for tests and full reporting of outcomes
☒	☐	Estimates of effect sizes (e.g. Cohen's *d*, Pearson's *r*), indicating how they were calculated

Our web collection on statistics for biologists contains articles on many of the points above.

Software and code

Policy information about availability of computer code

Data collection: No software was used to collect data.

Data analysis: AnnotSV (v3.4); BCFtools (v1.20); CADD score (v1.3); CADD-SV score (v1.1.2); Canu (v2.1.1); Clair3 (v1.0.2); Canvas (v1.40.0.1613+master); CNVnator (v0.3.3); cuteSV (v2.1.0); dbNSFP (v4.8a); deepTools (v3.5.5, https://github.com/deeptools/deepTools); DeepVariant (v1.4.0); Delly (v1.2.6); DESeq2 (v1.50.0); VEP (v110.1); GATK (v4.3.0.0); gnomAD (v4.1.0); hifiasm (v0.16.1); IGV (v2.16.0); Isoquant (v3.10.0); Jalview (v2.11.4.1); Kanpig (v0.3.1); Kraken2 (v2.1.3); LongPhase (v1.7.2); MAFFT (v7.525); Manta (v.1.5.0); merqury (v1.3); meryl (v1.4); methylink (v0.6.0, https://github.com/projectoriented/methylink); minimap2 (v2.28.0); Modkit (v0.3.1, https://github.com/nanoporetech/modkit); ntsm (v1.2.1); Paragragh (v2.4); PAV (v2.3.4); pbmm2 (v1.13.1, https://github.com/PacificBiosciences/pbmm2); PBSV (v2.9.0, https://github.com/PacificBiosciences/pbsv); RagTag (v2.1.0); rustybam (v0.1.33, https://github.com/mrvollger/rustybam); SAMtools (v1.16.1); Sawfish (v0.12.4); Smoove (v0.2.5, https://github.com/brentp/smoove); Sniffles (v2.2); Somalier (v0.2.19); subseq (https://github.com/EichlerLab/subseq-smk); SVbyEye R package; TRGT (v1.4.1); Truvari (v4.3.1); UCSC Genome Browser tracks; UCSC LiftOver; VerifyBamID (v2.0.1); yak (v0.1, https://github.com/lh3/yak.git).
Code and workflow developed in this manuscript: asap (https://github.com/EichlerLab/asap); BoostSV (https://github.com/jiadong324/BoostSV); contiguous-X (https://github.com/projectoriented/contiguous-X); continuous-methylation (https://github.com/projectoriented/continuous-methylation).

For manuscripts utilizing custom algorithms or software that are central to the research but not yet described in published literature, software must be made available to editors and reviewers. We strongly encourage code deposition in a community repository (e.g. GitHub). See the Nature Portfolio guidelines for submitting code & software for further information.

Data

Policy information about availability of data

All manuscripts must include a data availability statement. This statement should provide the following information, where applicable:

- Accession codes, unique identifiers, or web links for publicly available datasets
- A description of any restrictions on data availability
- For clinical datasets or third party data, please ensure that the statement adheres to our policy

The underlying sequencing data, as well as the processed assembly and alignment files used for analysis in this study for the SSC samples (n = 168) and the complete sample set (n = 189), are available to approved researchers through SFARI Base under Dataset ID DS0000104 (https://base.sfari.org/dataset/DS0000104) and through the National Institute of Mental Health Data Archive (NDA) under Collection ID 3780 (https://nda.nih.gov/edit_collection.html?id=3780). Source Data are provided with this paper.

Research involving human participants, their data, or biological material

Policy information about studies with human participants or human data. See also policy information about sex, gender (identity/presentation), and sexual orientation and race, ethnicity and racism.

Reporting on sex and gender	Sex was based on self-reported and genotypically assigned information.
Reporting on race, ethnicity, or other socially relevant groupings	Ancestry was predicted by Somalier (v0.2.19, Pedersen et al. 2020) using long-read sequencing data.
Population characteristics	SSC and SAGE: 174 individuals corresponding to 46 simplex families with unsolved autism (Turner et al. 2017, Wilfert et al. 2021).<br>Rett-like: 5 girls from 5 trios (15 individuals) with Rett-like syndrome, evaluated at Baylor College of Medicine, had no causal variants identified in MECP2 by prior gene panel testing or WES.
Recruitment	This study used previously collected datasets.
Ethics oversight	Ethical approval for this study was granted by the University of Washington IRB Committee B, under STUDY ID: STUDY00000383. This study is a data-driven computational analysis. We had no contact with study participants. All samples were encoded and anonymized.

Note that full information on the approval of the study protocol must also be provided in the manuscript.

Field-specific reporting

Please select the one below that is the best fit for your research. If you are not sure, read the appropriate sections before making your selection.

☒ Life sciences ☐ Behavioural & social sciences ☐ Ecological, evolutionary & environmental sciences

For a reference copy of the document with all sections, see nature.com/documents/nr-reporting-summary-flat.pdf

Life sciences study design

All studies must disclose on these points even when the disclosure is negative.

Sample size	A total of 51 autism simplex families (189 individuals).
Data exclusions	Data exclusion was performed based on availability and completion of data.
Replication	Samples were combined with three sets of population controls. Samples were also analyzed in two batches (with or without Rett-like cohort) and then combined into one batch.
Randomization	No known genetic cause was identified in the 51 probands from prior analyses. The 46 probands from SSC and SAGE do not exhibit exceptional polygenic risk scores. The selected families were predominantly those with female probands due to the interest in discovering X chromosome variants and the large-effect variants are more likely to be discovered.
Blinding	Blinding is not applicable to this line of analysis.

Reporting for specific materials, systems and methods

We require information from authors about some types of materials, experimental systems and methods used in many studies. Here, indicate whether each material, system or method listed is relevant to your study. If you are not sure if a list item applies to your research, read the appropriate section before selecting a response.

Materials & experimental systems

n/a	Involved in the study
☒	☐ Antibodies
☒	☐ Eukaryotic cell lines
☒	☐ Palaeontology and archaeology
☒	☐ Animals and other organisms
☒	☐ Clinical data
☒	☐ Dual use research of concern
☒	☐ Plants

Methods

n/a	Involved in the study
☒	☐ ChIP-seq
☒	☐ Flow cytometry
☒	☐ MRI-based neuroimaging

Plants

Seed stocks	*Report on the source of all seed stocks or other plant material used. If applicable, state the seed stock centre and catalogue number. If plant specimens were collected from the field, describe the collection location, date and sampling procedures.*
Novel plant genotypes	*Describe the methods by which all novel plant genotypes were produced. This includes those generated by transgenic approaches, gene editing, chemical/radiation-based mutagenesis and hybridization. For transgenic lines, describe the transformation method, the number of independent lines analyzed and the generation upon which experiments were performed. For gene-edited lines, describe the editor used, the endogenous sequence targeted for editing, the targeting guide RNA sequence (if applicable) and how the editor was applied.*
Authentication	*Describe any authentication procedures for each seed stock used or novel genotype generated. Describe any experiments used to assess the effect of a mutation and, where applicable, how potential secondary effects (e.g. second site T-DNA insertions, mosiacism, off-target gene editing) were examined.*
